# Flow-injection analysis for on-line monitoring of nutrients (ammonia and nitrite) in aquaculture

**DOI:** 10.1155/S1463924692000348

**Published:** 1992

**Authors:** A. C. Ariza, P. Linares, M. D. Luque de Castro, M. Valcárcel

**Affiliations:** Department of Analytical Chemistry Faculty of Sciences University of Córdoba Córdoba E 140004 Spain

## Abstract

This article describes photometric flow injection (FI) methods for the determination of ammonia and nitrite in aquaculture. The methods are based on the use of normal and reversed FI approaches and show the potential of this technique for monitoring the input and output streams of small tanks at young fish-breeding farms. The methods meet the requirements of fish hatcheries, particularly in terms of the high sampling rate allowable (40/h).


Journal of Automatic Chemistry, Vol. 14, No. 5 (September–October 1992), pp. 181–183

### Flow-injection analysis for on-line monitoring of nutrients (ammonia and nitrite) in aquaculture

**A. C. Ariza, P. Linares, M. D. Luque de Castro and M. Valcárcel**

*Department of Analytical Chemistry, Faculty of Sciences, University of Córdoba, E 140004 Córdoba, Spain*

*This article describes photometric flow injection (FI) methods for the determination of ammonia and nitrite in aquaculture. The methods are based on the use of normal and reversed FI approaches and show the potential of this technique for monitoring the input and output streams of small tanks at young fish-breeding farms. The methods meet the requirements of fish hatcheries, particularly in terms of the high sampling rate allowable (40/h).*

Ammonia is a nutrient which is transformed into nitrite or nitrate under bacterial action. Animals excrete ammonia with urea and peptides. Nitrite is an intermediate product from oxidation of ammonia or reduction of nitrate. High concentrations (>0·1 µat. $NO_2$-N/l) of nitrite in water are indicative of high bacterial activity. Both species require monitoring in fish farms because they must be strictly controlled, especially in farms which breed young fish. The permissible concentrations of these nutrients is higher as the fish age; so the frequency of the analysis is a function of the fish age.

Flow injection analysis (FIA) is an automatic technique which is highly suitable for on-line monitoring [1–6]. The aim of this research was to show the potential of FIA in aquaculture by developing the conventional batch methods usually used for monitoring in fish hatcheries in an automated unsegmented-flow fashion. The aim was to provide continuous on-line monitoring so that as soon as a parameter is out of the allowable range immediate corrective action can be taken. Two possibilities are proposed: (1) a normal flow injection (n-FI) method in which the sample is injected into a carrier which merges with the suitable reagents to form a coloured product which is measured at the wavelength of maximum absorbance; and (2) a reversed FI (r-FI) method in which the sample acts as carrier into which the reagent is inserted to obtain the coloured product to be monitored.

The two analytes chosen to show the potential of FIA in aquaculture are the ones that are most frequently determined in this field: nitrite and ammonia. Conventional, and well-established, derivatizing reactions were selected to obtain products easily measurable by a simple photometric detector. The foundation of the method for the determination of ammonia was the well-known Berthelot reaction [7] between the analyte and hypochlorite to form chloramine and then with phenol to yield indophenol blue, which was monitored at 625 nm. The determination of nitrite was based on the Griess reaction (Shinn modification [8] which avoids the use of carcinogenic reagents). Thus, the analyte reacts with sulphanilamide to form an azocompound which then reacts with N(1-naphthyl)ethylenediamine to yield the dye which shows maximal absorbance at 540 nm.

### Experimental

### *Reagents*

Hypochlorite solution: a 43·7 g/l aqueous solution of hypochlorite (from Carlo Erba) containing 0·48 g/l of sodium nitroprusside (from Merck). Phenol solution: 3 g of phenol (from Merck) were dissolved in ethanol: water 1:3 and adjusted to pH 12 with NaOH solution (from Merck) and them diluted to 1 l with the ethanol: water mixture. Sulphanilamide solution: 4 g of sulphanilamide (from Merck) were dissolved in 8 ml of concentrate hydrochloric acid (from Merck) for the normal FI method and in 4 ml of concentrate hydrochloric acid for the reversed FI method and then diluted to 1 l with distilled water in both cases. N-(1-naphthyl)ethylenediamine solution: 4 g/l (normal FI method) or 6 g/l (reversed FI method) of N-(1-naphthyl) ethylenediamite and 3% m/v (normal FI method) or 2·5% (reversed FI method) of sodium chloride (from Merck) were diluted to 1 l with distilled water. Buffer solution: the buffer solution contained 100 g/l of $NH_4Cl$, 20 g/l of $Na_2B_4O_7$, and 1 g/l of EDTA (from Merck). Ammonium and nitrite standard solutions of 1 g/l, prepared from ammonium sulphate and sodium nitrite, respectively (from Merck).

### *Instruments and apparatus*

A Unicam 8625 UV/Vis spectrophotometer equipped with a Hellma 178.12QS flow-cell (inner volume 18 µl) connected to a Knauer recorder was used. A Gilson Minipuls-3 peristaltic pump, a Rheodyne 5041 injection valve, and Tecator TMII chemifolds were also used. A 80286 compatible personal computer, equipped with a PC-ADDA/14 analogue-to-digital interface with 12 bit resolution for the data acquisition, a dual 'serial control' interface for the pump and valve multi input-output, a 40 MB hard disk and a 3½" floppy disk drive, and a STAR LC10 printer were also used.

### *Manifolds and procedures*

Figure 1 shows the arrangements used to implement the normal (A) and the reversed FI methods (B). For ammonia determination the sample was inserted into a basic stream to convert the ammonium ion into ammonia, which merges with the hypochlorite stream and then

0142–0453/92 $3.00 © 1992 Taylor & Francis Ltd.

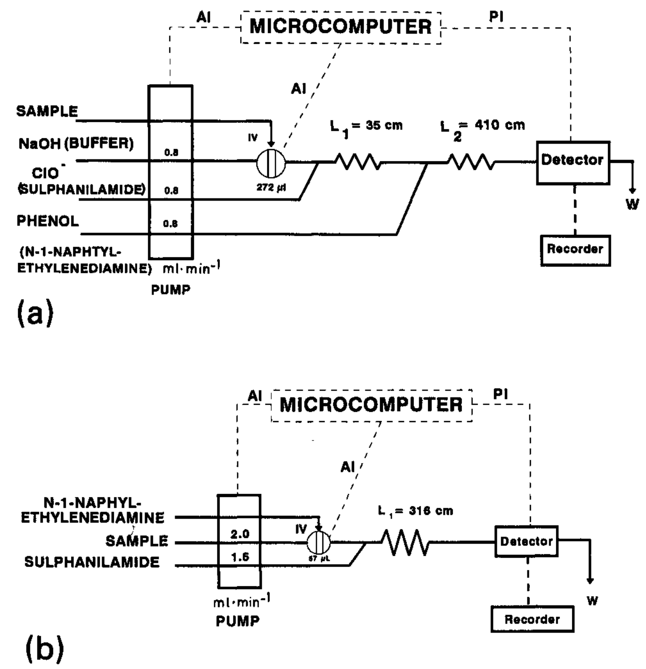

*Figure 1. Manifolds for determination of ammonia and nitrite by (a) normal FI mode, (b) reversed FI mode. IV denotes injection valve, AI and PI denote the active and passive interfaces, respectively; L the reactor and W waste.*

with the phenol stream to form indophenol blue, which is monitored at 625 nm on passage of the reacting plug through the flow-cell located at the photometric detector. The determination of nitrite by the normal FI method involves injecting the sample into the buffer stream, which merges with sulphanilamide to form the azocompound along $L_1$ and the final dye along $L_2$, after merging with N(1-naphythyl)ethylenediamine stream. The reversed method requires a simple manifold (see figure 1[*b*]), in which the sample circulates continuously through the main channel with which the sulphanilamide stream merges. The plug of N(1-naphthyl)ethylenediamine solution is injected when the concentration of nitrite is required.

### Results and discussion

### *Optimization of variables*

A detailed study of the variables affecting the performance of the proposed methods was performed in all instances. Variables were divided for these studies into chemical (concentration and pH of the different reagents) and FIA (flow-rate, injected volume and reactor lengths). They were all studied by the univariate method. The optimization was focused to achieve maximal sensitivity (height of the FI peak) with maximal sampling frequency and minimal injected sample or reagent volume. Table 1 shows the variables which were studied, the ranges in which they were investigated, and the optimal values found.

It is worth noting that the transient signals provided by the photometer in all methods were independent of the saline concentration in the samples, thus allowing the determination of the analytes in sea-water. This is important because a significant number of fish hatcheries feed their tanks with sea-water.

*Table 1. Study of the variables and optimal values.*

| Variable (nitrite determination) | Normal FIA Range studied | Normal FIA Optimal value | Reversed FIA Range studied | Reversed FIA Optimal value |
|---|---|---|---|---|
| Sulphanilamide concentration ($g\,l^{-1}$) | 2·0–6·0 | 3·8 | 2·0–8·0 | 4·0 |
| NaCl concentration (%) | 8·0–20·0 | 8·0 | 4·0–10·0 | 4·0 |
| N-(1-Naphthyl)ethylenediamine ($g\,l^{-1}$) | 1·6–6·0 | 4·0 | 2·0–8·0 | 6·0 |
| NaCl concentration (%) | 0·5–4·0 | 3·0 | 1·5–3·5 | 2·5 |
| Injection volume (µl) | 30–400 | 94 | 30–400 | 84 |
| Flow rate (ml $min^{-1}$) | 1·2–4·3 | 2·33 | 1·6–4·9 | 1·8 |
| Length reactor 1 (cm) | 45–200 | 50 | 200–400 | 315 |
| Length reactor 2 (cm) | 60–300 | 100 | — | — |

| Variable (ammonia determination) | Normal FIA Range studied | Normal FIA Optimal value |
|---|---|---|
| Phenol concentration ($g\,l^{-1}$) | 2·0–4·0 | 3·0 |
| NaOH concentration ($g\,l^{-1}$) | 10·30 | 20·0 |
| Hypochlorite ($g\,l^{-1}$) | 15–58 | 43·7 |
| Nitroprusside ($g\,l^{-1}$) | 0·12–0·72 | 0·48 |
| Injection volume (µl) | 100–450 | 272 |
| Flow rate (ml $min^{-1}$) | 0·9–3·0 | 2·38 |
| Length reactor 1 (cm) | 35–200 | 35 |
| Length reactor 2 (cm) | 200–500 | 410 |

### *Features of the methods*

Once the variables were optimized, the optimum values found were used to run calibration curves over a wide range of concentration of each analyte to determine the linear portion of each method. Table 2 shows the features of these methods. The sensitivity was acceptable in all instances, and the determination ranges cover the concentrations required for monitoring these analytes in fish hatcheries and small tanks. Precision was also acceptable. The anlytes can be determined by a triplicate insertion of each sample at a rate of 30 samples per hour.

A study of the possible interferents was performed for each analyte. As can be seen in table 3, almost all of the common species in these systems tolerate a high analyte/foreign ion ratio. This makes the method highly suitable for application to the proposed systems.

### *Application of the methods to real samples*

The proposed methods were applied to the determination of the analytes in six different fish feeds consisting of fish flavour and insect larvae of different texture and grain size, which were suspended in tanks containing tap-water ($F_1$ to $F_3$ in figure 2) and sea-water ($F_4$ to $F_6$ in figure 2).

*Table 2. Features of the methods.*

| | Mode | Equation | *r* | RSD | Linear range ($\mu g\ ml^{-1}$) |
|---|---|---|---|---|---|
| Nitrite | n-FIA | $H = 0{\cdot}279 + 0.047\ \vert NO_2^-\vert$ | 0·999 | ±3·48 | 0·5–8·5 |
| | r-FIA | $H = 0{\cdot}032 + 0{\cdot}483\ \vert NO_2^-\vert$ | 0·999 | ±0·96 | 0·1–2·0 |
| Ammonia | n-FIA | $H = 5{\cdot}7{*}10^{-3} + 0{\cdot}0012\ \vert NH_4^+\vert$ | 0·998 | ±1·04 | 5–80 |

*Table 3. Study of foreign species in the determination of nitrite and ammonia.*

| Tolerated ratio foreign ion : $NO_2^-$ | Foreign ion |
|---|---|
| 100 : 1 | $Na^+$, $K^+$, $NH_4^+$, $Ca^{2+}$, $Mg^{2+}$, $Hg^{2+}$, $Cu^{2+}$, $Cl^-$, $NO_3^-$, $Co_3^{2-}$, $SO_4^{2-}$, $SiO_3^{2-}$ |
| 75 : 1 | $PO_4^{3-}$, $Na^+$, $K^+$, $Ca^{2+}$, $Hg^{2+}$, $NO_3^-$, $CN^-$, $SCN^-$ |
| **Tolerated ratio foreign ion : $NH_4^+$** | **Foreign ion** |
| 100 : 1 | $Na^+$, $K^+$, $Ca^{2+}$, $Hg^{2+}$, $NO_3^-$, $CN^-$, $SCN^-$ |
| 25 : 1 | Glycine |
| 20 : 1 | $S^{2-}$ |
| 10 : 1 | $Cu^{2+}$ |
| 5 : 1 | Urea |

The contents of the tanks was continuously monitored every 30 min for two weeks. The values shown in table 2 are an average of the measurements performed during the first 6 hours each day. The concentrations of ammonia and nitrite increased over the period of monitoring as a consequence of the fish-feed degradation.

### Conclusions

The proposed methods allow ammonia and nitrite in tap-water and sea-water samples to be monitored with a high degree of automation for long unattended periods; hence they provide a means for the routine daily determination of these parameters in fish farms.

The methods afford automatic on-line determination, which is of great interest for the analytical monitoring of the input and output streams of water tanks. The ease and rapidity with which the results can be obtained allows immediate corrective action to be taken.

### Acknowledgements

The authors wish to thank the Comisión Interministerial de Ciencia y Tecnología (CICyT) for financial support (under grant No. MAR88-0112).

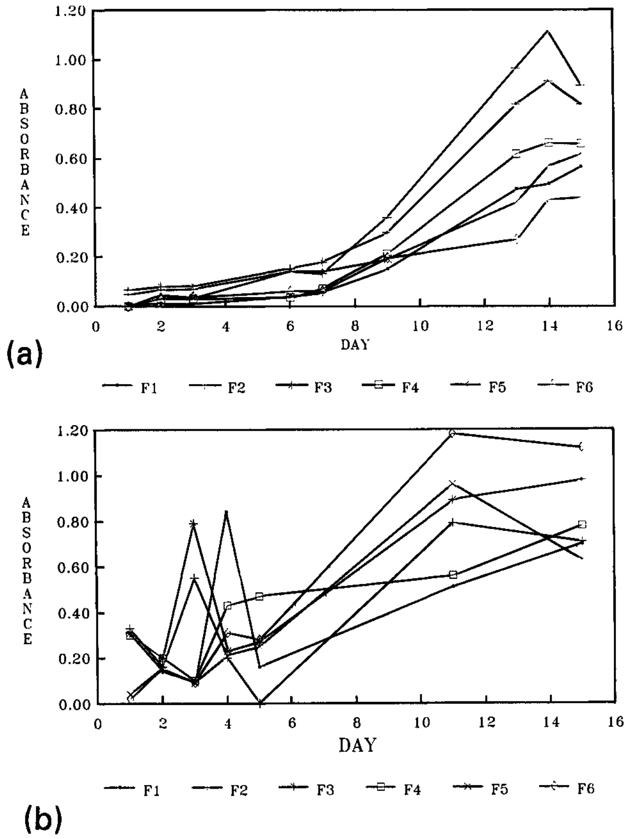

*Figure 2. Evolution of the ammonia (a) and nitrite (b) content from fish-feed suspended in tap-water (F1 to F3) and sea-water (F4 to F6).*

### References

1. Bayer, Th., Herold, Th., Hiddessen, R. and Shügerl, K., *Analytica Chimica Acta,* **190** (1986), 213.
2. Moseur, X. and Motte, J. C., *Analytica Chimica Acta,* **204** (1988), 127.
3. Appelqvist, R., Johansson, G., Holst, O. and Mattiasson, B., *Analytica Chimica Acta,* **216** (1989), 299.
4. Gisin, M. and Thommen, C., *Trends in Analytical Chemistry,* **8** (1989), 62.
5. Luque de Castro, M. D., *Talanta,* **36** (1991).
6. Daunert, S., Bachas, L. G., Ashcom, G. S. and Meyerhoff, M. E., *Analytical Chemistry,* **62** (1990), 318.
7. Berthelot, M. E. P., *Report de Chemie Appliqué,* **284**, 859.
8. Shinn, M. B., *Ind. Eng. Chem. Anal. Ed.,* **13** (1941), 33.

